# The Potential Effect of Polysaccharides Extracted from Red Alga *Gelidium spinosum* against Intestinal Epithelial Cell Apoptosis

**DOI:** 10.3390/ph16030444

**Published:** 2023-03-15

**Authors:** Marwa Ajala, Mickael Droguet, Marwa Kraiem, Hajer Ben Saad, Zakaria Boujhoud, Abderraouf Hilali, Hatem Kallel, Jean Marc Pujo, Ibtissem Ben Amara

**Affiliations:** 1 Laboratory of Medicinal and Environment Chemistry, Higher Institute of Biotechnology, University of Sfax, Sfax 3029, Tunisia; 2 ORPHY, Optimization of Physiological Regulation, Faculty of Medicine and Health Sciences, 29238 Brest, France; 3 Laboratory of Health Sciences and Technologies, High Institute of Health Sciences, Hassen University, Casablanca 20000, Morocco; 4 Intensive Care Unit, Cayenne General Hospital, Cayenne 97300, French Guiana; 5 Tropical Biome and Immunopathology, CNRS UMR-9017, Inserm U 1019, University of Guyane, Cayenne 97300, French Guiana; 6 Emergency Department, Cayenne General Hospital, Cayenne 97300, French Guiana

**Keywords:** intestinal epithelial cells, apoptosis, *Gelidium spinosum*, antioxidant, polysaccharides

## Abstract

Gut injury is a severe and unpredictable illness related to the increased cell death of intestinal epithelial cells (IECs). Excessive IEC apoptotic cell death during the pathophysiological state entails chronic inflammatory diseases. This investigation was undertaken to assess the cytoprotective action and underlying mechanisms of polysaccharides from Tunisian red alga, *Gelidium spinosum* (PSGS), on H_2_O_2_-induced toxicity in IEC-6 cells. The cell viability test was initially carried out to screen out convenient concentrations of H_2_O_2_ and PSGS. Subsequently, cells were exposed to 40 µM H_2_O_2_ over 4 h in the presence or absence of PSGS. Findings revealed that H_2_O_2_ caused oxidative stress manifested by over 70% cell mortality, disturbed the antioxidant defense, and increased the apoptotic rate in IEC-6 cells (32% than normal cells). Pretreatment of PSGS restored cell viability, especially when used at 150 µg/mL and normal cell morphology in H_2_O_2_-callenged cells. PSGS also equally sustained superoxide dismutase and catalase activities and hindered the apoptosis induced by H_2_O_2_. This protection mechanism of PSGS may be associated with its structural composition. The ultraviolet visible spectrum, Fourier-transformed infrared (FT-IR), X-ray diffraction (XRD), and high-performance liquid chromatography (HPLC) demonstrated that PSGS is mainly sulfated polysaccharides. Eventually, this research work provides a deeper insight into the protective functions and enhances the investment of natural resources in handling intestinal diseases.

## 1. Introduction

The blooming growth in the world population along with changes in the level of lifestyle and eating habits have been crucial in the advent of multiple diseases constituting a severe threat to human life [1]. Inflammatory bowel diseases (IBDs) are chronic idiopathic diseases marked by relapsing gastrointestinal tract inflammation [2]. Bloody diarrhea, abdominal pain, hepatosis, and eye and skin lesion are the main clinical symptoms of these diseases. IBDs involve two forms, ulcerative colitis (UC) and Crohn’s disease (CD) [3]. CD represents the more profound, transmural, inflammatory condition in patches throughout the small intestine and the colon. Meanwhile, UC is marked by mucosal inflammation confined to the colon [4].

IBDs constitute a global and significant public health challenge that is increasing in newly industrialized countries, especially those in Africa, Asia, and South America [2]. They impact over 3.6 million people in the United States and Europe [5]. The precise etiology of IBDs remains unknown, but scientists concluded the presence of complex interactions of genetic, dysregulated immune responses, environmental factors, destruction of the intestinal barrier, and potentially other risk factors [6]. Yet, the epithelial barrier is generated by a monolayer of specialized intestinal epithelial cells (IECs) that are intrinsic in terms of preventing the passage of pathogens, toxins, and allergens from the gastrointestinal lumen into the circulatory system [7]. Therefore intestinal barrier destruction raises intestinal permeability, destroys immune system homeostasis, and produces inflammatory responses, apoptosis, and oxidative stress [8,9].

According to the World Health Organization, IBD has been referred to as one of the modern refractory diseases. Thus, conventional treatments are largely faced with the problem of cellular tolerance to drugs making treatments ineffective and triggering numerous side effects as well as irreversible organ damage around the target site [10], the risk of vomiting, infections, abdominal pain, and malignancies [11]. From this perspective, it is fundamental to seek an alternative functional-food–based approach in a safer, more productive, and cheaper for preventing or effectively handling IBD.

So far, biologically natural products have been deemed to be treasured for various biological activities, such as immunomodulatory [12], antioxidant [13], anti-inflammatory [14], and pharmacological [15] activities owing to their outstanding curative effects and low toxicity [16]. Seaweeds, from which biologically active polysaccharides are isolated, can also be attributed to specific products of natural origin. Polysaccharides are important biological macromolecules generated by the dehydration and condensation of more than 10 monosaccharide molecules. These substances exhibit promising physiological activities, likely immunomodulatory, anti-inflammatory, anti-oxidation, hypoglycemic, antibiotic, and antitumor agents [17], and other beneficial properties, such as anti-hyperlipidemia [18], antiviral [19], antiradiation [20] and anticoagulant activities [21]. Their features refer to their structure and physicochemical characteristics, relying on the organism they are produced from. Accumulating evidence confirms that polysaccharides play a vital role in treating acute colitis and display a powerful potential to enhance current IBD treatment [3]. Marine algal polysaccharides can also scavenge ROS and improve the antioxidant system to ease intestinal inflammation [22]. The red macroalga genus *Gelidium* is mainly known as the source of carrageenan. The variability of the primary structure of carrageenans determines the diversity of their macromolecular organization and determines a wide range of their biological activity [23], including anti-cancer [24], immunomodulatory, anticoagulant, antithrombotic, antiviral, and antitumor effects [25]. Due to their biocompatibility, high molecular weight, high viscosity, and gelling capacity, these polymers have gained great importance in recent decades not only in the food industry, but also in medical, pharmaceutical, and biotechnological research [26], cosmetics, printing, and textiles [27]. Approximately 20% of the largest agar industrial sources come from *Gelidium* spp. [28]. *Gelidium spinosum* was studied for the first time in Tunisia under the name of *Gelidium latiforium* in 1999 to explore some hydro biological elements and agar-potentiality. However, there remains limited information regarding its phytochemical constituents and its potential biological activities [29].

The main objective of this experiment is to extract sulfated polysaccharides from *Gelidium spinosum* and to evaluate their structure characterization and protective effects against H_2_O_2_-induced oxidative damage and apoptosis in IEC6 cells as related mechanisms, as well as to provide an experimental basis for the high-value utilization of PSGS.

## 2. Results and Discussion

### 2.1. Extraction Yield

Polysaccharides correspond to polar macromolecules, which are easy to dissolve in water since they can replace water-water interactions with water-solute interactions. According to Nai et al. [30], polysaccharides extraction yield is influenced by extraction parameters. PSGS extraction yield was measured relying on the wet weight of alga powder. The yield rate of 18.32% proved to be better than the previously reported one for polysaccharides extracted from several other algae, including *Gelidium crinale* (2.6%) [31] and *Chondrus canaliculatus* (2.05%) [32]. However, it turned out to be lower than other red seaweeds, such as *Gracilaria gracilis* (30.0%) [33].

### 2.2. Chemical Analysis

The quantitative estimation of PSGS revealed a significant contribution of carbohydrates and a lower amount of uronic acid and proteins. Proteins are part of a cell wall structure and are associated closely with polysaccharides. It has been considered as a potential contaminant of polysaccharides [34]. After depigmentation and extraction of PSGS, we attempted to denature proteins and eliminate the majority of lipids. For this reason, we acquired relatively low levels of proteins (4.81%) compared to other polysaccharides [35], which is indicative of the extraction method [36]. Amounts of proteins depend mainly on the method of extraction and deproteination processes. Fleury et al. [37] asserted that the precipitation of proteins during extraction at 100 °C contributes probably to their indigestibility. The PSGS protein contents were similar to those from the endodermis of shaddock [38]. However, the results presented in Table 1 revealed that PSGS had relatively high total sugar levels (67.28%).

On the other side, the uronic acid content of PSGS (14.30%) was similar to that obtained by polysaccharides extracted from *Sargassum vulgare* (brown alga) [39]. The marine origin, seasonal periods, conditions, and extraction method are determining factors for the variations of all these contents.

The ash contents were estimated at a percentage of 2.64 ± 0.41%. As reported by Rioux et al. [40], the proportion of minerals can be the result of the association between polysaccharides and cations or the inorganic salt in the water absorbed by seaweed. Concerning sulfate esters, the chemical analyses demonstrated amounts of 17.30%, similar to those found in polysaccharides of red seaweed *Gelidium pacificum* [41].

### 2.3. Structural Characterization

#### 2.3.1. UV and FT-IR Spectroscopy Analysis

**Figure 1:**
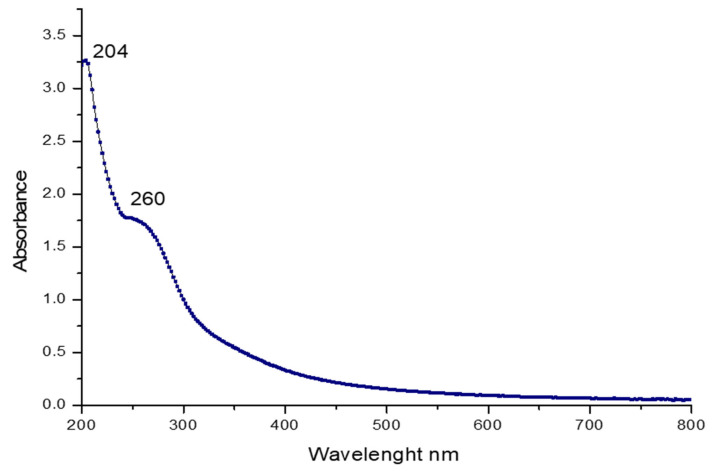
UV–visible absorption Spectrum of polysaccharide PSGS.

Figure 1 illustrates the UV spectra of PSGS. The data observed in Figure 1 display two peaks. The first corresponds to a broad absorption around 204 nm, confirming that PSGS is specified as polysaccharides. Jose et al. [42] indicated a significantly prominent absorbance peak at 205–215 nm using sulfated polysaccharide from brown seaweed *Padina tetrastromatica*. The second slight absorption peaks at 260–280 nm indicate the presence of proteins [43].

**Figure 2:**
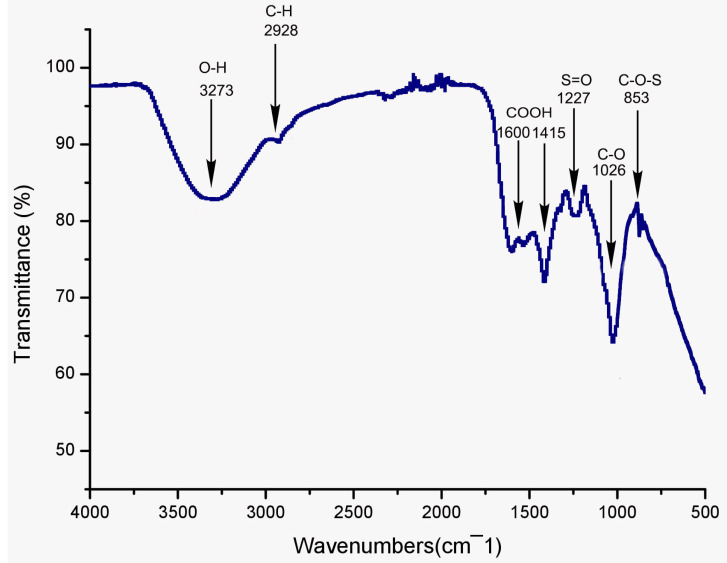
IR spectrum of the polysaccharide PSGS.

As it is well known, FT-IR spectroscopy stands as a handy tool to identify the structural features of polymer blends, such as distinct organic groups in the polysaccharide. The infrared spectroscopy results in Figure 2 revealed that PSGS had typical polysaccharide absorption peaks in the region between 400 and 4000 cm^−1^. The broad absorptions around 3273 cm^−1^ and 2928 cm^−1^ were attributed to O-H stretching vibration and C-H stretching vibration of the -CH- groups, respectively [44], indicating that the sample was a polysaccharide compound. The asymmetric and symmetric vibration of the carboxylate groups appeared at around 1600 cm^−1^ and 1415 cm^−1^, respectively, demonstrating that PSGS was an acidic polysaccharide [45], implying in turn the presence of uronic acids, which was confirmed by monosaccharide composition. Both hydroxyl and carboxyl groups played an intrinsic role in the biological activities of polysaccharides. Previous studies [46] unveiled that extracellular polysaccharide containing carboxyl and hydroxyl groups can enhance their antitumor and antioxidant activities. This polysaccharide has an absorption peak at 1538 cm^−1^, revealing its content of proteins [47], which was proven by UV analysis. The absorption band at 1227 cm^−1^ was attributed to the S=O stretching vibration [48]. The absorption peak at 1026 cm^−1^ was assigned for the presence of glycosidic linkage stretch vibration of C–O bond in guluronic units [32]. Combined with an absorption band at 853 cm^−1^, originating from the C-O-S stretching vibration [49], the three bands suggest the existence of sulfate in the PSGS, which was corroborated by chemical analyses. Positive specific rotation and the characteristic absorption at 853 cm^−1^ indicated the α-configuration of the sugar units [50].

#### 2.3.2. X-ray Diffractometry (XRD) Analysis

**Figure 3:**
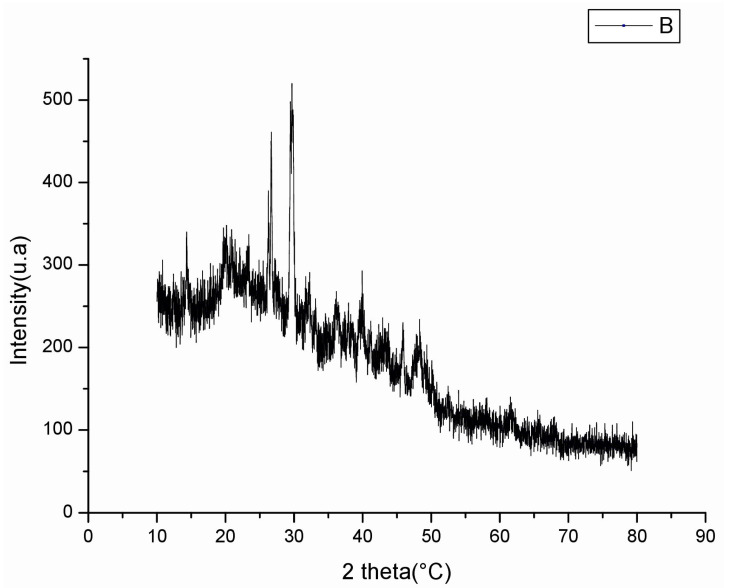
X-Ray Diffraction of PSGS.

Numerous polysaccharides correspond to bioactive products, and their biological activities are closely related to their structural characteristics, such as monosaccharide composition, glycosidic bonds, and crystalline structure. Figure 3 illustrates the X-ray diffractogram of PSGS ranging between 0° and 100°. Data demonstrated a major crystalline reflection at 29°, and PSGS tends to be a semi-crystalline polymer. Crystalline and semi-crystalline structures of materials were directly influenced by various physical properties, including tensile strength, flexibility, solubility, swelling, viscosity, or opaqueness of the bulk polymer [51].

#### 2.3.3. Monosaccharide Composition Analysis

**Figure 4:**
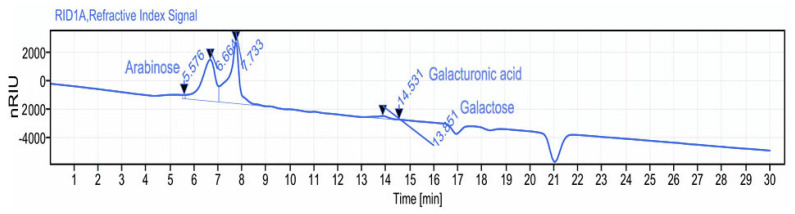
Monosaccharide composition analysis by HPLC−FID of PSGS.

The biological activities of polysaccharides are strongly affected by their monosaccharide composition. Previous data indicate that algae represent a rich source of monosaccharides [52,53]. In the current work, PSGS monosaccharide composition was investigated through HPLC-FID analysis. The latter displayed a heterogeneous behavior, where arabinose, glucuronic acid, and galactose constitute the major monosaccharide units at retention times of 5.57; 14.53, and 13.65 min, respectively, according to the elution time of monosaccharide standards (Figure 4). Previous data regarding polysaccharides extracted from green macroalga *Chaetomorpha linum* also revealed heterogeneous compositions of monosaccharides [54]. Referring to the literature, extraction protocol, temperature, and solvent used for precipitation affect the nature of molecules and extraction yield [55].

### 2.4. Effect of PSGS and H_2_O_2_ on IEC-6 Cells Viability

IEC-6 cells were stimulated with different concentrations of PSGS (20; 50; 70; 100; 150, and 200 µg/mL) for 12 h, and the cell viability was determined by MTT assay.

**Figure 5:**
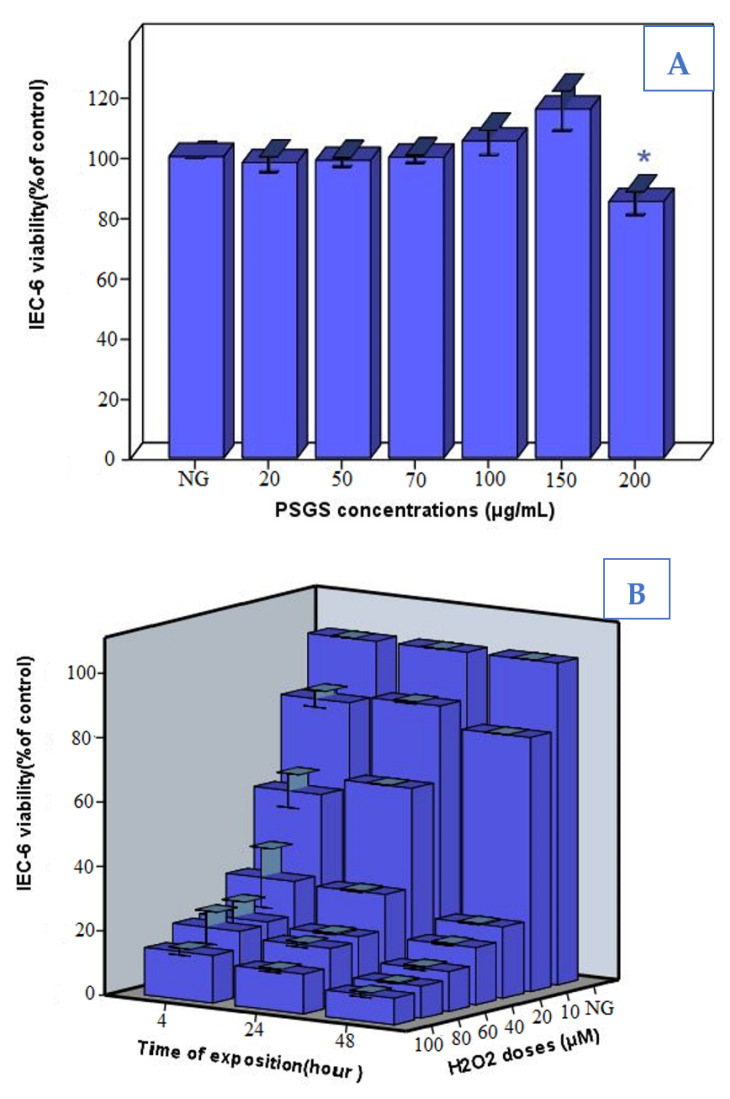

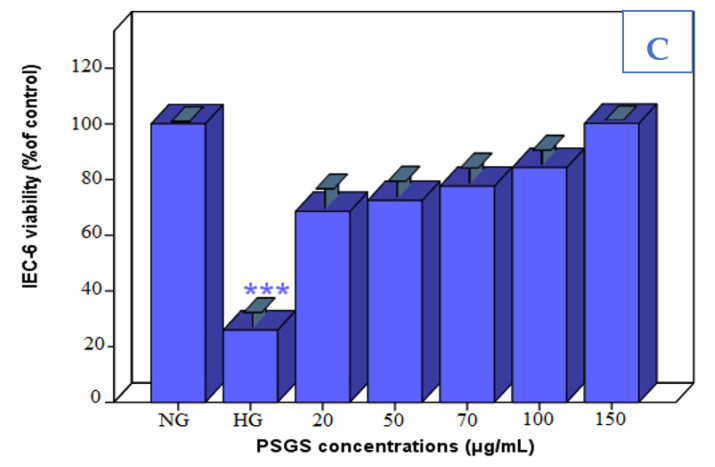
(**A**) Toxicity test of PSGS on cell viability of IEC-6 cells (% of control), (**B**) Effects of H_2_O_2_ on cell viability of IEC-6 cells (% of control), (**C**) Effects of PSGS on cell viability in H_2_O_2_-injured IEC-6 cells (% of control).NG (Normal group) HG (treated group with H_2_O_2_) Error bars represent standard deviations of three replications. Bars marked with (*) are significantly different from the normal group at * *p* < 0.05; *** *p* < 0.001.

As displayed in Figure 5A, cell viability increased with increasing PSGS concentrations. Our findings agree with those reported in the study of Qiu et al. [56], which indicated that natural polysaccharides from red seaweed *Porphyra haitanensis* are nontoxic to IEC-6 cells and promote at the same time cell proliferation. Previous studies emphasized that antioxidant capacities and beneficial effects of natural compounds might be inversed and become lethal for the cells under such conditions as high concentrations [57]. At 200 µg/mL, the cell survival rate reached 15% compared to the control group (*p* < 0.05), which might damage cells, therefore indicating a dose-dependent relationship between viability rate and concentrations of PSGS.

In our experimental model of cellular oxidative damage, we stimulated IEC-6 cells with different concentrations of H_2_O_2_ for 4, 24, and 48 h exposure duration. Exposure concentration significantly altered the cell viability of IEC-6 cells in a dose/time-dependent manner (Figure 5B). Besides, cell growth was remarkably inhibited, departing from 40 µg/mL by 70% and decreased dramatically to reach 90 % to 80 and 100 µM H_2_O_2_. Based on these results, IEC-6 cells were treated with 40 µM H_2_O_2_ for 4 h as an oxidative damage model in the present study. Bettaib et al. [58] selected 40 µM H_2_O_2_ for 4 h as a stress condition in IEC-6 cells to assess the cytoprotective effect of phenolic compounds.

The viability of IEC-6 cells exposed to PSGS and H_2_O_2_ was investigated through MTT analysis. Our results (Figure 5C) demonstrated a significant difference between the control group and the model (H_2_O_2_) group, suggesting that the survival rate of IEC-6 cells significantly decreased after 4 h of H_2_O_2_ stimulation. However, pre-incubation of 20; 50; 70; 100, and 150 μg/mL PSGS, for 24 h, significantly increased the survival rate of IEC-6 cells. PSGS were collectively beneficial in protecting IEC-6 cells against H_2_O_2_-induced injury. Meanwhile, it was inferred that the sulfate group is highly related to free radicals, including superoxide and hydroxyl scavenging effects. This can further explain why PSGS showed significant improvement in the viability of IEC-6 cells. Thus, sulfate polysaccharides display an outstanding protective ability to handle the adverse effects of H_2_O_2_, which is consistent with a previous report [59].

### 2.5. Effect of PSGS and H_2_O_2_ on the Morphological Aspect

**Figure 6:**
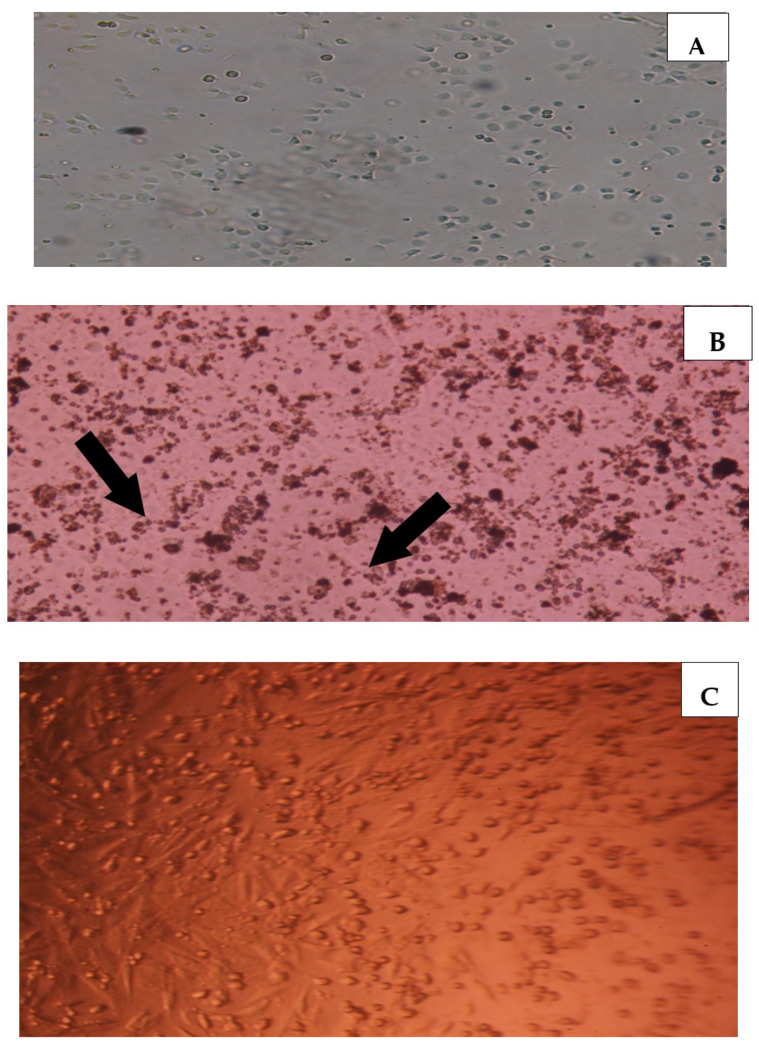
Photomicrographs demonstrating morphological changes of IEC-6 cells for different treatments (**A**) normal; (**B**) model (H_2_O_2_ 40 µM,4 h arrows indicating changes in cell shape); (**C**) pretreated with PSGS 20 µg/mL for 4 h then exposed to 40 µM of H_2_O_2_ for 4 h.

To further explore the protective effects of PSGS on IEC-6 cells, morphological changes were examined under an inverted microscope. As exhibited in Figure 6B, observing IEC-6 cells under the inverted photonic microscope demonstrated impressive changes in cell shape. H_2_O_2_ crosses the membrane and interferes with cell attachment to initiate cellular damage, such as cell shape changes and mitochondrial dysfunction, leading to metabolic alterations [58,60]. In addition, a high number of dead cells in response to H_2_O_2_ toxicity was observed. Referring to previous studies [61], the time of incubation as well as the dose of H_2_O_2_ tightly influence the survival rates of IEC-6 cells. However, cells co-treated with both H_2_O_2_ and PSGS showed a morphology close to that of control cells, suggesting the protective effect of PSGS against the toxicity induced by H_2_O_2_.

### 2.6. PSGS Supported Enzymatic Defense against H_2_O_2_ Toxicity

**Figure 7:**
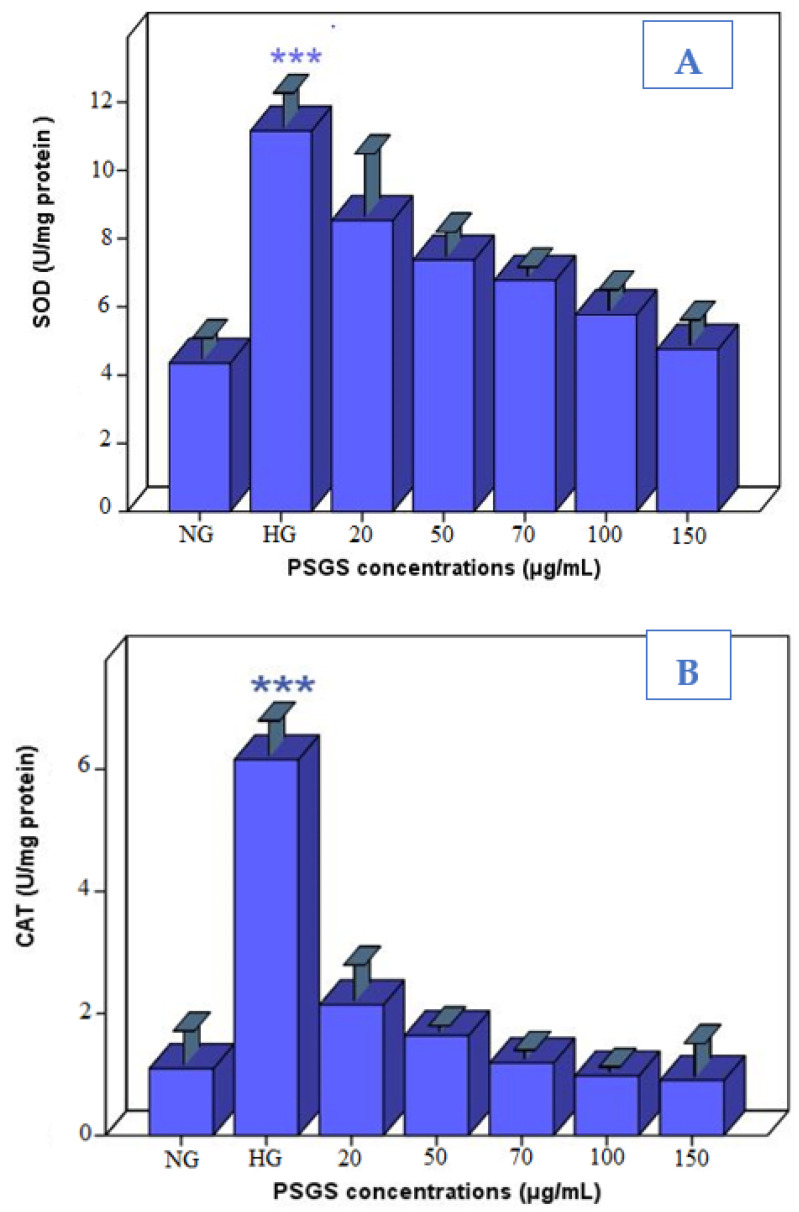
(**A**) Effects of PSGS on levels of SOD in H_2_O_2_-injured IEC-6 cells, (**B**) Effects of PSGS on levels of CAT in H_2_O_2_-injured IEC-6 cells. NG (normal group), HG (model group treated with H_2_O_2_). Error bars represent standard deviations of three replications. Significant differences between the treated groups and the normal group were mentioned as follows: *** *p* < 0.001.

The organism balances pro- and antioxidant systems in response to oxidative stress conditions. As an important index for detecting oxidative cell damage, SOD corresponds to an antioxidant enzyme catalyzing superoxide anions’ dismutation to H_2_O_2_ and O_2_ [22]. CAT is a common antioxidant enzyme that utilizes oxygen and catalyzes the degradation or reduction of H_2_O_2_ to water and molecular oxygen. Consequently, it completes the detoxification process initiated by SOD [62]. Compared to the control group, the activity of SOD in IEC-6 cells increased after treatment by 40 µM of H_2_O_2_ for 4 h (Figure 7A). It is to be noted that, the SOD activity in PSGS-treated cells gradually decreased according to the PSGS concentrations. The result indicates the dose-dependent relationship between SOD activity and polysaccharides concentrations. Overproduction of ROS, including superoxide, singlet O_2_−, and hydroxyl radical, was the chief cause of oxidative damage and cell apoptosis [63]. Based on the mechanism stated above, several native sulfated polysaccharides isolated from red algae were also found to increase the SOD activity [64] remarkably.

However, the binding effect of CAT activity was achieved at 150 µg/mL of PSGS (Figure 7B). Therefore, the protective capacity of these promising polymers against H_2_O_2_-induced oxidative stress might reside under their antioxidant actions through enhancing endogenous antioxidant enzyme activities. Evidence indicated that polysaccharides containing uronic acid have significant antioxidant activity owing to carboxyl groups, which behaved as important electron or hydrogen donors in the antioxidant activity [65].

### 2.7. Effect of PSGS on H_2_O_2_ Induced Apoptosis in IEC-6 Cells

**Figure 8:**
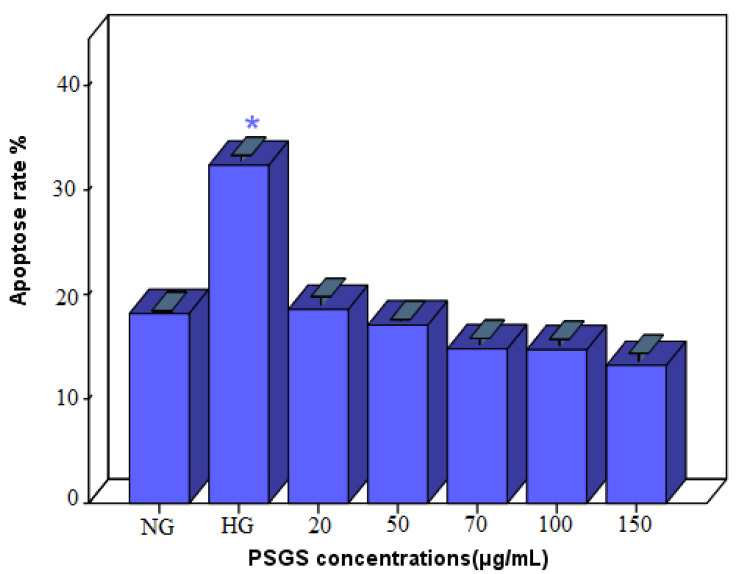
Effect of PSGS on H_2_O_2_-induced apoptosis. NG (normal group) HG (treated group with H_2_O_2_). Error bars represent standard deviations of three replications, Significant differences between the treated groups and the control were mentioned as follows: * *p* < 0.05.

Apoptosis is a fundamental and crucial biological phenomenon that plays an intrinsic role in clearing abnormal cells [66]. Thus, IECs renewal is necessary for maintaining tissue homeostasis. Still, excessive IEC cell death disrupts intestinal barrier integrity and permits the invasion of luminal antigens into the lamina propria, a hallmark of intestinal inflammation [67]. H_2_O_2_ is a membrane-permeable ROS generator that is widely used to induce oxidative damage and apoptosis in cells [47]. Sound evidence revealed that oxidative stress causes programmed cell death [68]. As far as our study is concerned, IEC-6 cells were stained with Oxazole Yellow, and apoptotic cells were computed using fluorescence microscopy. As plotted in Figure 8, cells treated with H_2_O_2_ alone exhibited a higher apoptosis rate of 32% than normal cells, indicating that H_2_O_2_ induced apoptosis. Zhuang et al. [69] indicated that 40 ng/mL H_2_O_2_ induces apoptosis in Chondrocytes. As expected, the cell apoptosis rate decreased after pretreatment with PSGS, suggesting that the PSGS can effectively mediate oxidative damage and protect IEC-6 cells against H_2_O_2_-induced apoptosis. Different concentrations of polysaccharides displayed more potent anti-apoptotic effects. Thus, due to their marked effect on cell viability, the pretreatment with 150 µg/mL of polysaccharides displayed a lower percentage of apoptosis (13.2 ± 0.64). Previous studies revealed that oxidative stress and apoptosis are detected in many diseases and are caused by an imbalance between free radical generation and antioxidant defense capacity [70]. Therefore, the results suggest that PSGS can protect endothelial cells from apoptosis. At this stage of analysis, it is noteworthy that polysaccharides from *Gelidium spinosum* might be considered as promising molecules in terms of injury recovery and degenerative diseases referring to their anti-apoptotic capacities. For a deeper and better understanding of the effect of polysaccharides on apoptotic cell death, Ma et al. [68] highlighted that sulfated polysaccharides can effectively mediate oxidative damage and significantly protect PC12 cells against H_2_O_2_-induced apoptosis.

## 3. Materials and Methods

### 3.1. Chemicals and Reagents

Rat-derived intestinal epithelial cell line (IEC-6 cells) was purchased from Public Health England (888071401). Dulbecco’s Modified Eagle’s Medium (DMEM), an antibiotic mixture (100 µg/mL of streptomycin and 100 UI/mL of penicillin), fetal bovine serum (FBS), trypsin/EDTA, and trypan blue were obtained from Lonza (Ploermel, France). Phosphate buffered saline (PBS) was afforded by Dominique Dutscher (Bernolsheim, France). Hydrogen peroxide 30% (*w*/*w*) was supplied by MERCK Laboratories (Darmstadt, Germany). Dimethyl sulfoxide (DMSO), 3-(4,5-dimethylthiazol-2-yl)-2, (-diphenyl tetrazolium bromide (MTT) was purchased from Sigma Aldrich (Schnelldorf, Germany). Oxazole Yellow for apoptosis as well as detection kits for superoxide dismutase (SOD) and catalase (CAT) were purchased from CliniSciences.

### 3.2. Seaweed Collection and Processing

**Figure 9:**
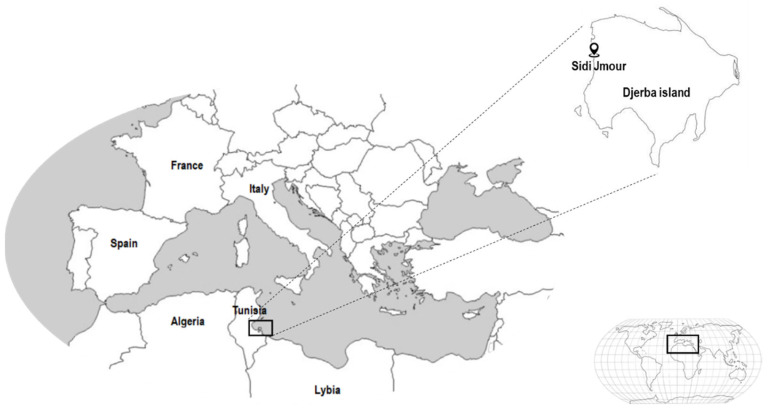
Location of the collection zone (the coastal area of Sidi Jmour, Djerba, Tunisia).

The red alga Gelidium spinosum was collected in March 2021 from the coastal area of Sidi Jmour, Djerba, Tunisia. Google maps coordinates are (33°51′22.4″ N 10°44′33.4″ E). Gelidium spinosum was authenticated by a specialist in ecology, Professor “Asma Hamza”, accredited with the World Register of Marine Species (WoRMS) under the following identifier “145594” (Figure 9)

Collected seaweed was washed thoroughly to remove surface impurities, salt, and sand particles, and epiphytes. The water was drained off, and the seaweed sample was dried in the dark. The dried seaweed was powdered in the grinder and preserved in a limp sterile for further studies.

### 3.3. Polysaccharides Extraction

The polysaccharide extraction procedure was performed according to the method reported by Gong et al. [71], using hot water for extraction and ethanol as a precipitating agent. Notably, the seaweed flour (50 g) was dispersed in distilled water, stirred at 90 °C for 4 h, and filtered. The filtrate was centrifuged at 3600× *g* for 10 min. After centrifugation and concentration, the ethanol was incorporated (V/3V) to a concentration of 95% for alcohol precipitation at 4 °C for 24 h. The crude polysaccharide (PSGS) was obtained after centrifugation using a refrigerated centrifuge and lyophilization. The yield was expressed in terms of the ratio of the dry weight of the polysaccharide extracted (g) against the dry weight *Gelidium spinosum* (g) in percentage. The dried PSGS was stored at −20 °C for further studies.

### 3.4. Chemical Characterization of PSGS

#### Determination of Total Carbohydrate, Protein, Uronic Acid, Sulfate, and Ash Content

As reported by Huang et al., carbohydrate content was quantified using the phenol sulfate acid method [72]. Basically, 0.1 mg/mL standard glucose solution was prepared; 0.1, 0.2, 0.4, 0.6, 0.8, and 1.0 mL were pipetted in a test tube. Next, 1 mL of distilled water and 1 mL of 3% phenol were added. Afterward, 4 mL of concentrated sulfuric acid was inserted gradually. The absorbance was measured at 490 nm after reaction for 30 min at room temperature.

Soluble proteins in PSGS were quantified by colorimetric assay [73]. The content of the uronic acid was assessed through the use of the Carbazole-sulfate method [74]. Notably, the galacturonic acid solution was configured similarly and served as a standard. In an ice bath, 6 mL of superior pure sulfuric acid were added, shaking while adding. Subsequently, 0.2 mL of 0.1% carbazole-ethanol (25 mg carbazole dissolved in 25 mL ethanol) was added, and the reaction was carried out for 2 h. The absorbance was measured at 530 nm.

Sulfate content was determined according to the gelatin-barium method [75], using 1 mg/mL of potassium sulfate (K_2_SO_4_) as standard.

The amount of ash contents in PSGS was measured according to the method reported by Seedevi et al. [76]. In brief, 0.5 g of the dried polysaccharides taken in a porcelain crucible was burnt at 550 °C for 8 h in a muffle furnace. The weight of the residue, which represents the ash content, was recorded and the results are given as percentage of the dry weight of polysaccharides.

### 3.5. Structural Characterization of PSGS

#### 3.5.1. Ultraviolet and Fourier Transform Infrared (FT-IR) Spectroscopic Analysis

The ultraviolet spectrum of PSGS was recorded using an UV-vis spectrophotometer (JENWAY/7315, Staffordshire, UK) in the 200–800 nm range.

The infrared spectrum of PSGS was determined on a Nicolet FT-IR spectrometer. The spectrum was acquired at a resolution of 4 cm^−1^, and the measurement range was 4000–400 cm^−1^ at room temperature. OPUS data collection software program was next used to analyze the data (Bruker, Ettlingen, Germany) [43].

#### 3.5.2. X-ray Diffractometry (XRD) Analysis

An X-ray diffractogram of PSGS was recorded using an X-ray diffractometer (D8 advance, Bruker, Bremen, Germany). The data were obtained in the 2θ ranges 5–80° with a step size of 0.05° and a counting time of 5 s/step.

#### 3.5.3. Monosaccharide Composition Analysis

HPLC-FID recorded the Monosaccharide composition of PSGS according to the method described by Xie et al. [77]. A five-milligram sample was dissolved within 3 mL of 2 mol/L TFA and hydrolyzed at 110 °C for 3 h. Subsequently, TFA was removed by washing with methanol.

Hence, 50 mg of sodium tetrahydruroborate were added to reduce the hydrolyzed product. Then, pyridine and acetic acid anhydride were added at 40 °C for 2 h to acetylation. The acetylated sample was filtered and analyzed by HPLC-FID.

Galactose, glucose, fructose, galacturonic acid, glucuronic acid, arabinose, and xylose were used as standard sugars.

### 3.6. Cytoprotective Activity of PSGS on IEC-6 Cells

#### 3.6.1. Measurement of IEC-6 Cells Viability

Cell viability was determined using an MTT assay. IEC-6 cells were seeded into a 96-well plate at 2 × 10^4^ cells/well. After incubation in 5% of CO_2_ at 37 °C for 24 h, different concentrations of PSGS ranging from 20–200 µg/mL were inserted into the well for co-culturing during 24 h. 150 µL of 0.05% MTT solution was added for 2 h. Then, 100 µL of DMSO was added to dissolve the produced formazan prior to incubation with IEC-6 cells for 30 min. This procedure was followed by measuring the absorbance at 540 nm using a microplate reader.

Different concentrations (10–100 μM) of H_2_O_2_ were used to stimulate IEC-6 cells for 4, 24, and 48 h in 96-well plates to estimate a suitable level for the cell injury model.

The cytoprotective effect of PSGS was then evaluated. IEC-6 cells were incubated with different concentrations of PSGS for 24 h and then stimulated with 40 µM H_2_O_2_ for 4 h. The obtained results are indicative of the mean of three independent experiments. Cell viability is suggestive of the absorbance of treated groups relative to that of the control group.

#### 3.6.2. Cell Morphology Observation

After each treatment, as depicted above, cell morphology was examined with an inverted microscope (Olympus Optical, Rungis, France).

### 3.7. Determination of Antioxidant Enzymes Activity

IEC-6 cells were placed in a Petri dish at 2 × 10^4^ cells/well. Then, they were exposed to 40 µM H_2_O_2_ for 4 h, both with and without PSGS at different concentrations (20; 50; 70; 100; 150 μg/mL). After treatment, cells were rinsed with ice-cold PBS, scraped, and sonicated at 4 °C. After homogenization and centrifugation for 10 min at 4 °C, protein concentration was determined in the supernatant according to the method of Bradford [78]. Finally, samples were stored at −80 °C for subsequent analysis.

#### 3.7.1. Superoxide DISMUTASE Activity

SOD activity was measured using a commercially available kit (SOD activity Elabscience, Houston, TX, USA). The principle of the method relies on the ability of SOD to neutralize superoxide ions created by the xanthine/xanthine oxidase system and subsequently inhibit the reduction of WST-1 (water-soluble tetrazolium salt) to WST-1 formazan. In this respect, IEC-6 cells obtained 24 h after oxidative stress induction were washed with ice-cold 1× PBS and lysed, as described in the kit protocol. The supernatant of each sample was collected, and the total SOD activity was assayed spectrophotometrically at 450 nm. SOD concentration, expressed in units per milligram of protein, was specified using the SOD standard curve.

#### 3.7.2. Catalase Activity

CAT activity was estimated using a commercially available kit (CAT activity Elabscience, Houston, TX, USA). The principle of the method rests on the ability of CAT to decompose hydrogen peroxide. Ammonium molybdate can stop this reaction, and the residual H_2_O_2_ reacts with ammonium molybdate to generate a yellow complex. The production of the yellow complex which can calculate CAT activity at 405 nm and CAT concentration, and which is expressed in units per milligram of protein, was determined using the SOD standard curve.

### 3.8. Apoptosis Rate Detection

The apoptosis assay was carried out using a fluorescent cyanine Oxazole Yellow (YP1). YP1 does not penetrate the plasma membrane of viable cells. However, during apoptosis, apoptotic processes cause the cell membrane to become slightly permeable. This allows Oxazole Yellow (YP1) to enter these cells and bind to nucleic acids so as to detect apoptotic cells. Notably, IEC-6 cells were collected after washing with PBS and digesting with trypsin. Then, they were centrifuged to keep the cells in suspension. Next, 50 µL of Oxazole Yellow were added and mixed. The cells were then cultured at 20–25 °C for 5–10 min and protected from light. The apoptosis cells observed under fluorescence microscopy represent small, bright green dots.

## 4. Conclusions

To sum up, a sulfated polysaccharide was successfully extracted from the red seaweed *Gelidium spinosum*. This study evaluated the protective effect of PSGS on H_2_O_2_-induced injury cells. The result indicates that the PSGS pre-incubation not only hinders oxidative stress in intestinal epithelial cells, but also inhibits H_2_O_2_-induced apoptosis by scavenging ROS.

Similar to other active polysaccharides, this study prompts that the PSGS may have an important value to prevent and cure oxidation-related diseases, and it may be well applied in medical health care in the future.

Finally, it is worth noting that more diligent efforts should be performed in this area to further explore the functions and mechanisms of PSGS in relation to intestine function through additional in vitro and in vivo studies.

## Figures and Tables

**Table 1:** The chemical composition of PSGS.

Content %	PSGs
Total sugars	67.28 ± 0.08
Proteins	4.81 ± 0.06
Uronic acids	14.30 ± 0.13
Sulfate	17.30 ± 0.77
Ash	2.64 ± 0.41

## Data Availability

Data is contained within the article.
